# Planning “Plan B”: The Case of Moving Cattle From an Infected Feedlot Premises During a Hypothetical Widespread FMD Outbreak in the United States

**DOI:** 10.3389/fvets.2019.00484

**Published:** 2020-01-09

**Authors:** Emily Walz, Jessica Evanson, Fernando Sampedro, Kimberly VanderWaal, Timothy Goldsmith

**Affiliations:** ^1^Department of Veterinary and Biomedical Sciences, College of Veterinary Medicine, University of Minnesota, St. Paul, MN, United States; ^2^Center for Animal Health and Food Safety, College of Veterinary Medicine, University of Minnesota, St. Paul, MN, United States; ^3^Division of Environmental Health Sciences, School of Public Health, University of Minnesota, Minneapolis, MN, United States; ^4^Department of Veterinary Population Medicine, College of Veterinary Medicine, University of Minnesota, St. Paul, MN, United States

**Keywords:** foot and mouth disease, FMDV, carcass, cattle, feedlot

## Abstract

In the event of a foot-and-mouth disease (FMD) outbreak in the United States, “stamping out” FMD infected premises has been proposed as the method of choice for the control of outbreaks. However, if a widespread, catastrophic FMD outbreak in the U.S. were to occur, alternative solutions to stamping out may be required, particularly for large feedlots with over 10,000 cattle. Such strategies include moving cattle from infected or not known to be infected operations to slaughter facilities either with or without prior implementation of vaccination. To understand the risk of these strategies, it is important to estimate levels of herd viremia. Multiple factors must be considered when determining risk and feasibility of moving cattle from a feedlot to a slaughter facility during an FMD outbreak. In addition to modeling within-herd disease spread to estimate prevalence of viremic animals, we explore potential pathways for viral spread associated with the movement of asymptomatic beef cattle (either pre-clinical or recovered) from an infected feedlot premises to offsite harvest facilities. This analysis was proactive in nature, however evaluation of the likelihood of disease spread relative to disease (infection) phase, time of movement, and vaccination status are all factors which should be considered in managing and containing a large-scale FMD outbreak in the United States.

## Introduction

Foot and mouth disease (FMD) is a highly contagious viral disease affecting primarily cloven-hoofed animals. The disease is characterized by the development of vesicles in and around the mouth and on the feet. Although natural FMD infection rarely causes death of mature animals, the disease results in decreases in livestock productivity and causes serious economic impacts on international trade of animals and animal products (1). FMD was last reported in the United States (U.S.) in 1929 and in North America in 1952 (Canada) and 1954 (Mexico). In the event of an FMD incursion in the US, the US Department of Agriculture Foreign Animal Disease Preparedness and Response Plan (FAD PReP) Red Book likely will be followed (2). This response plan details activities for outbreaks at various scales and geographies. Historically, “stamping out” has been the preferred tool, however if the FMD outbreak is at an endemic scale (Type 4: Widespread or National FMD Outbreak or Type 5: Catastrophic FMD Outbreak), other strategies proposed by the FAD PReP Strategy Document such as vaccinate-to-live and vaccinate-to-slaughter likely will be considered (3). At present, identification of FMD within a herd is reliant upon observation of clinical signs to trigger diagnostic testing of suspect individuals. This lack of population-level disease surveillance testing methods results in delayed detection until infection has spread at the farm level.

The US has a number of large-scale livestock production facilities. If one of these operations were infected, depopulation on-site likely is not practical. Instead, options for the management of these animals are needed if they are not depopulated, including use of vaccination before moving animals, allowing disease to progress through a herd before movement, and moving vaccinated animals from not-known to be infected premises to decrease local susceptible population. We use FMDV infection at a large scale cattle feedlot (over 10,000 head) to highlight some of the potential risks and considerations that decision-makers may factor into their response plans in the event of an FMD outbreak.

## Predicting Disease Spread Throughout the Herd

The shedding phase of FMD is the time interval between the time an animal begins shedding virus to the time an animal is no longer shedding virus and it generally includes pre-clinical and clinical infectious phases. A carrier phase is also possible; this includes animals that have recovered from clinical disease and have at least one positive esophageal-pharyngeal sample 28 days or more post infection (4). While hypothetically plausible, transmission of FMDV from carrier cattle to susceptible individuals has never been conclusively documented (5).

**Figure 1:**
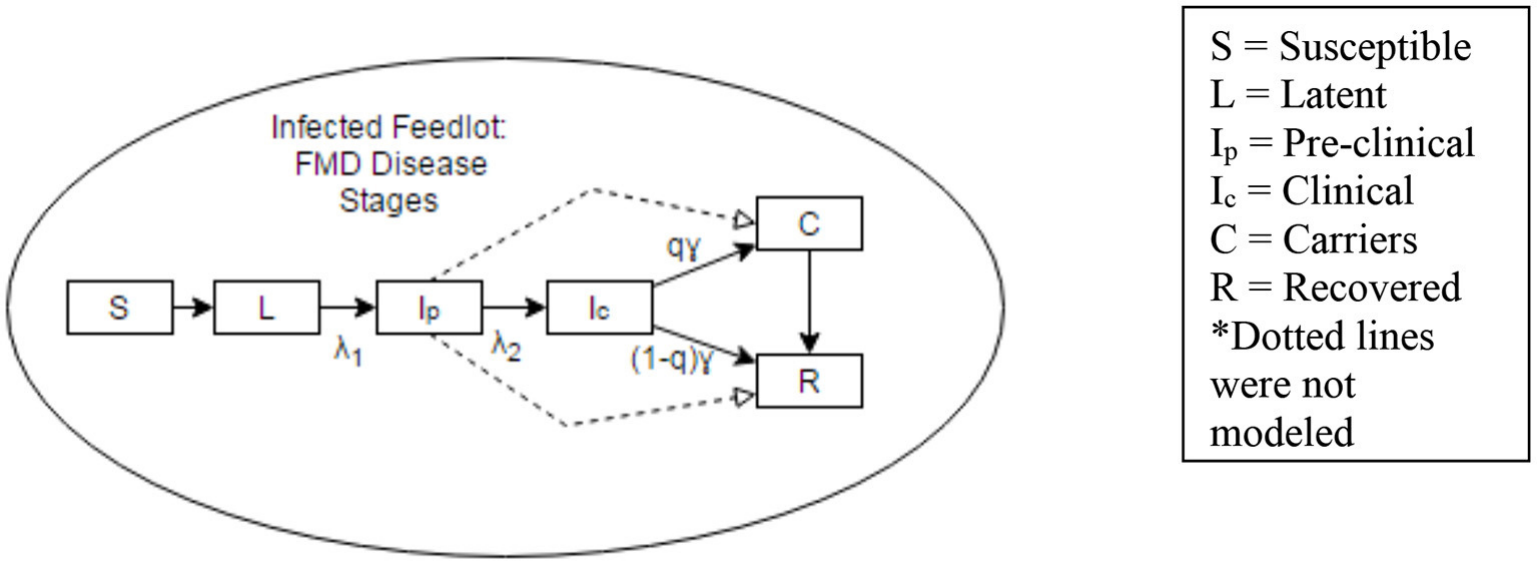
Representation of disease states considered in a within-herd disease spread model. Dotted lines were not considered in the model.

Five different hypothetical FMD management scenarios were explored. In order to estimate the number of cattle in each of the disease phases, a within herd disease spread model was developed and applied to the scenarios. This model used a 10,000 head beef cattle herd to determine the number of cattle in susceptible (S), latent (L), pre-clinically infectious (I_p_), clinically infectious (I_C_), carrier (C), and fully recovered (R) disease phases at different times over a time period of 65 days (Figure 1). This time period of 65 days was chosen based on the recovery time (i.e., no more infectious individuals) predicted by the model for a 10,000 head cattle herd. The main output of the model was the proportion of cattle in different phases of infection at different time points. The periods of time that would present the highest likelihood of virus transmission when shipping cattle to slaughter were then identified.

The model updates the number of cattle in each disease state every 24 h, which provides insight into the disease progression through the herd. The model considers the uncertainties in input parameters as well as the inherent variability associated with the course of infection in each animal and the spread within the group. Parameter distributions for the disease spread model were obtained from previous work (6–8). Additional information on model structure, parameters and inputs can be found in Supplementary Material.

The scenarios evaluated were:

- *Scenario 1: The disease is allowed to progress through an infected herd and at least 42 days have passed since the day clinical signs were initially detected prior to movement of asymptomatic cattle at or near target market weights to harvest*.
- *Scenario 2: The feedlot is actively infected (animals with clinical signs are present) and cattle not showing clinical signs of FMD (non-infected, latent, viremic non-clinical, recovered) that are at or near target market weights are moved to harvest without a waiting period*.
- *Scenario 3: Upon detection, all cattle in the infected feedlot are vaccinated, at least 42 days have passed since the day clinical signs were initially detected in the herd and asymptomatic cattle at or near target market weights are subsequently moved to harvest*.
- *Scenario 4: Upon detection, all cattle in the infected feedlot are vaccinated, at least 14 days have passed as the waiting period post-vaccination and cattle not showing clinical signs of FMD (non-infected, latent, viremic non-clinical, recovered) that at or near target market weights are moved to harvest*.
- *Scenario 5: The feedlot is not known to be infected (infected but undetected or negative) and is located within a Control Area. All animals have been vaccinated and cattle at or near target market weights are moved to harvest after a 14-day waiting period*.

## The Role and Impact of Vaccination

In situations where emergency vaccination is authorized, animals that get vaccinated are those not showing clinical signs of infection. Various vaccination strategies to control outbreaks and restore disease-free status have been employed in outbreaks in the Netherlands (9) and in South America (10). In experimental studies, if animals are sufficiently and adequately immunized by vaccination before virus challenge, both within-herd transmission and the likelihood of between-herd transmission will decrease (11–13). Emergency vaccination with high-potency vaccines against FMDV has been shown to be highly effective in preventing clinical signs in animals when the correct type and strain are used in the vaccines and when the vaccine was administered no fewer than 4 days prior to challenge with FMDV (14–17).

While vaccinated animals appear to be protected from clinical infection, sub-clinical infection may occur. Viral RNA titers are, on average, 100–1,000 times lower (two to three log reduction) in the positive samples of vaccinated animals compared with those of the unvaccinated animals, suggesting vaccination can help reduce the amount of viral shedding into the environment shortly after direct virus exposure (15). Other findings suggest that vaccination helps to significantly reduce clinical signs in cattle and prevent viremia (18, 19). On a herd-level, as the time between vaccination and virus challenge increases, it is likely that the proportion of animals showing clinical signs in a herd will decrease (18).

Some proportion of cattle may become persistently infected regardless of their vaccination status (20). Cox et al. (15) showed that 45% of vaccinated cattle became persistently infected where much of the virus persists within the oropharynx and/or pharyngeal fluid (21). Viral persistence may also be influenced by the amount of virus an animal was exposed to, as well as the type of vaccine itself (16).

It is assumed that unvaccinated cattle produce significantly higher quantities of virus and continue to excrete virus for longer periods of time relative to vaccinated cattle (22). This may impact the amount of virus found in the environment at the infected or recovered premises. The environmental viral load also may depend on how many animals were able to develop adequate immunity prior to virus exposure.

To date, most experimental studies focus on cattle that have been vaccinated at least 3 days prior to challenge with FMDV (23). This is in contrast to the proposed use of emergency vaccination in a herd in which FMD has already been detected. It can be assumed that during vaccination of infected herds, there may be animals in all stages of disease (e.g., naïve, latent, sub-clinical, clinical, recovered). For response purposes, it is also prudent to emphasize the importance of vaccinating nearby susceptible premises prior to moving animals off of infected premises. Identifying the effects of vaccines on viral shedding in animals that are exposed to the virus prior to inoculation is an area for further research.

## Transportation Timelines and Moving Animals From an Infected Feedlot

The decision to move asymptomatic (e.g., uninfected, latent, viremic non-clinical, and recovered) cattle from an FMD infected or recovered premises may be influenced by logistics, finances, risk tolerance, and other factors. While other destinations may be used in the event of an outbreak, the movement of cattle was modeled from feedlot premises to harvest only; movements to other types of facilities were not considered.

In a large cattle herd, it is assumed that ~10% (1,000 cattle in a 10,000 head herd) of the herd will show clinical signs before the disease is detected. This could represent a worst-case scenario as the disease detection will be delayed due to inability for personnel to identify animals showing clinical signs until large numbers of lame animals are noted (24). It is predicted that the time until FMDV first detection would be ~17.5 days (95% CI = 17.4–17.7) after disease introduction to the premises.

In the absence of vaccination, delaying movement until most of the herd has reached the recovered stage is one strategy to decrease virus spread. Scenario 1 estimates time until recovery for an individual animal at 42 days, however, when considering movement of a large herd, waiting an additional 6 days would result in a lower likelihood of disease transmission from infected animals. 96% (9,628/10,000) of a beef cattle herd of 10,000 head will have entered the recovered phase at 65.7 days after disease introduction to the premises (95% CI = 65.3–65.9) resulting in a viremic population of 0.009% (0.942/10,000) given a 48 day waiting period from time of detection. Of note, some proportion of animals in the recovered stage may have healing or scarred lesions remaining. While these animals do not represent a virus transmission risk, they may not be eligible for shipment until all lesions have resolved.

If unvaccinated, asymptomatic cattle are moved more quickly after disease detection (Scenario 2), there is a risk that asymptomatic cattle moved may include individuals in a viremic pre-clinical disease state. Additionally, a larger proportion of viremic (pre-clinical and clinical) cattle still remain in the herd, adding to virus contamination in the feedlot environment. After a waiting period of 25 days post-detection, most cattle have moved into clinical or recovered states; ~0.012% (1.2/10,000) of pre-clinical cattle are predicted to be present in the herd. Moving all cattle at once likely is not feasible, due to limited capacity of transportation resources and slaughter plant capacity. In this work, we provide a point-in-time proportion of cattle in each disease state in the overall herd. The model did not account for decreasing overall herd size as transports to market are ongoing, however as the waiting time progresses past 25 days, the likelihood of transporting pre-clinical animals decreases. This number, however, does not include the number of sub-clinical cattle that may be present in the herd and shedding a similar amount of FMDV as clinical cattle. Literature estimates that ~11% of infected cattle may remain sub-clinical (25) as cited in Sutmoller and Olascoaga (26). The model did not account for sub-clinical cattle and this is a limitation of this methodology.

Vaccination status may have an effect on the likelihood of disease spread. Emergency vaccination of cattle has been shown to be effective in preventing or reducing clinical disease, reducing intra-herd transmission, and decreasing FMDV shedding. However, unlike the majority of experimental studies where animals had been vaccinated 3 or more days prior to disease exposure or challenge, the scenarios presented in this analysis involve the vaccination of cattle that may have already been exposed to the virus on an infected premises. Cattle that were vaccinated prior to exposure have been shown to remain carriers for a shorter period of time and harbor significantly less virus than cattle that were not vaccinated (22). It is unknown whether this applies to animals that are vaccinated after exposure to the virus. Vaccination after exposure may result in more viral shedding and more carriers being present than if the animals had been vaccinated prior to exposure.

If all cattle were vaccinated upon detection and movement was delayed until nearly the entire herd reaches the “recovered” state (Scenario 3), the primary concern for virus shedding is cattle in the “carrier” state. Similar to Scenario 1, 48 days post-detection represents the time at which 96% of a 10,000 head beef cattle herd was predicted to be in the recovered phase. The proportion of cattle in a carrier state when vaccination occurred very close to or after virus exposure is not known.

Cattle that have been vaccinated have been shown to have less severe or no clinical signs, and decreased viral shedding. There may also be some evidence to indicate fewer of these animals become sub-clinically infected (18). If FMDV was detected on day 17 and cattle were immediately vaccinated (Scenario 4), movement of these animals could occur no sooner than 31 days after initial infection (i.e., after the 14 day vaccine withdrawal period for slaughter). On day 31, ~0.34% (34/10,000) of the herd will be in the latent phase, and ~1.59% (159/10,000) of the herd will be in the pre-clinically infectious phase. Should a decision be made to move the eligible cattle on day 31 post-infection, there would be a larger likelihood of moving pre-clinical cattle relative to the longer waiting periods used in Scenarios 1 and 3.

If the feedlot is not known to be infected, it may truly be uninfected, or the level of clinical disease may be below the rate of detection (Scenario 5). Infection may occur any time before or after vaccination. The model predicts that it will take 17 days post-infection to reach 10% of the given population showing clinical signs of infection; thus FMDV will most likely not be detected in this herd prior to transport at the end of a 14-day vaccine withdrawal period for slaughter, resulting in the movement of infectious animals. A decision to move animals 14 days after vaccination (i.e., maximum 14 days after the herd was infected), represents a worst case scenario. Movement at this time could result in a large number of viremic pre-clinical animals being included in the transport. At Day 14, ~3% (343/10,000) of the herd would fall in this category.

## Risks to Neighboring Premises and Epidemiological Contacts

During an FMD outbreak, if a large feedlot operation is marketed early via transportation of animals to slaughter, the benefits of decreasing the local susceptible population must be weighed against the potential risks. Potential risks to surrounding premises and contacts include mechanical or aerosol transmission from movement of viremic animals or from the use of contaminated transport vehicles.

Pre-clinical and clinical cattle can shed virus in a variety of excretions and secretions, including high viral titers in nasal discharge, upper respiratory tract samples, skin lesions, probang (oropharyngeal) samples, and aerosolized virus, and to a lesser extent in urine and feces (27, 28). In contrast, carrier state cattle shed intermittently only from the esophageal-pharyngeal region (22). To date, there is no evidence that carrier cattle are capable of transmitting FMDV to susceptible animals, thus we assume transmission from these animals is not likely (5). The possibility of virus traveling via fomites on cattle hides, vehicle conveyance, or aerosolized in transit is possible, but further research in this area is needed to quantify the associated risk.

The absolute impact of vaccination after virus exposure on decreasing carrier state frequency or duration is unknown. Vaccination can, perhaps, be assumed to result in the possibility of lower levels of virus in the environment due to an assumed decrease in viral shedding from vaccinated animals.

Loading and transporting cattle from a feedlot that is currently infected means increased likelihood of contact with more virus in the environment when compared to a recovered feedlot, due to the presence of viremic animals that are actively shedding virus. While we assume that all transport vehicles will be cleaned and disinfected before and after each load of cattle, the risk for contamination of the vehicle may still remain. Additional logistical concerns may arise and include lack of washing facilities, waste water management concerns, and temperature and weather challenges. While these are outside the scope of this assessment, decision-makers should consider their potential impacts on viral spread.

## Limitations

While scenarios and models such as those used in this analysis are useful for proactive disease outbreak response planning, they may not correlate exactly with the parameters that arise during a real outbreak. Data from previous outbreaks detailing within-herd spread is limited (29); in absence of outbreak data, this model was based on characteristics described in experimental work, where only limited numbers of animals and few virus strains have been studied. Differences in virus characteristics during an outbreak, therefore may vary. It was also assumed that all animals with adequate virus exposure would progress to clinical disease. Lack of consideration for potential animals in the sub-clinical state is a limitation of this design. Before applying the findings of any prospective work to an actual outbreak, parameters and assumptions of a model must be assessed and contextualized if work such as this is considered for use in decision-making.

The model also assumed that all animals at a feedlot had the potential for contact with one another, which is likely not true in a majority of the commercial feedlot industry in the US Cattle in the feedlot are grouped by lots and pens, and while some mixing may occur at entry and sort dates, this contact is neither homogenous or random. The decision to use a simplified homogenous random-contact model was based on the lack of within-herd spread data available from past outbreaks (29) and findings from a similar study in swine where simplification to a homogenous mixing model did not significantly change key outcomes (30). In that study modeling FMD spread on a swine operation compared within-farm transmission assuming homogeneous mixing of a closed population and compared it to transmission when features of farm structure, demography and movement were incorporated into the model. They found that the assumption of homogeneous mixing in a closed population may be sufficient when considering the mean time to detection of a herd based on the presence of clinical signs in post-weaning pigs (30).

Future work is needed to evaluate if this holds true for a beef feedlot setting. The model employed in this work did not account for variations in management structures, husbandry practices, feedlot set-ups or sizes which may all impact contact rates and lameness detection capabilities. Decreased contact rates between sub-populations in a large herd may significantly increase the amount of time needed for FMD to spread (and for animals to recover) on a premises-level, making this work somewhat of a “best-case” scenario with rapid spread. The role for aerosol spread between pens has also not yet been thoroughly investigated in a field setting. Conversely a larger proportion of lame animals in a given pen may occur sooner than that same proportion of lameness is noted premises-wide. In the event that the infected pen is one under intensive scrutiny (e.g., at/near market weight or other intensive husbandry processing procedure) this may speed detection by clinical signs, while if the pen remains relatively unobserved detection may be delayed. Finally, while the use of vaccination was considered, we did not account for potential delays due to the time required to manufacture, ship, and administer vaccines in an identified positive herd.

## Conclusion

Incident managers tasked with managing large cattle operations during an FMD outbreak likely will consider many factors in deciding when and how to move potentially infected animals from an infected premises, including mechanism of spread, disease phase, time of movement, and vaccination status. It is likely that a single control strategy such as stamping out will be inadequate in the event that large feedlots become infected. When considering alternatives, the role of vaccination, especially when it is administered very close to or after viral exposure, remains unclear. Within-herd spread models such as the one used here are limited in the ability to represent all aspects of a potential outbreak. They may, however, be used as a step toward understanding the potential disease spread characteristics during an FMD outbreak, including the potential benefits of delaying movement on a known infected premises until clinical disease has resolved in the herd. Future work may incorporate new data as it arises, such as within-herd sub-populations, and changing more complex herd dynamics. During an outbreak, it is unlikely that just-in-time calculations will be available based on outbreak disease parameters. Proactive models may assist incident managers in gauging the likelihood that a load of cattle contains viremic and shedding animals (pre-clinical or carrier disease states), which pose greatest risk to other livestock premises. All proactive work must be reviewed for validity and applicability to a specific disease scenario at the time when it arises, and it is of great importance that proactive work such as this be interpreted in the context of available data and science as well as the assumptions and limitations. Overall, risks and benefits of moving asymptomatic cattle from an infected premises must be carefully assessed, as our results indicate there is no zero-risk period for moving cattle during disease progression through a herd.

## Data Availability Statement

All datasets generated for this study are included in the article/Supplementary Material.

## Author Contributions

TG and FS led project development and provided oversight for this work. FS and KV formulated and developed the modeling components for this study. JE participated in drafting initial reports and literature review. EW updated previous work and wrote the manuscript. All authors reviewed and provided critical feedback on the manuscript before submission.

### Conflict of Interest

The authors declare that the research was conducted in the absence of any commercial or financial relationships that could be construed as a potential conflict of interest.
