# Supplementary material for: Planning “Plan B”: The Case of Moving Cattle From an Infected Feedlot Premises During a Hypothetical Widespread FMD Outbreak in the United States
Source: Front Vet Sci. 2020 Jan 9;6:484. doi: 10.3389/fvets.2019.00484 (PMC6964524; doi:10.3389/fvets.2019.00484)
Supplement: Supplementary file 1 [file Data_Sheet_1.docx]

**Supplemental Material Online**

**Model inputs, parameters and assumptions**

This model used a 10,000 head beef cattle herd to determine the number of cattle in susceptible (S), preclinically infectious (I_p_), clinically infectious (I_C_), carriers (C) and fully recovered (R) disease phases at different times over a time period of 65 days. The model updates the number of cattle in each disease state every day. The uncertainties in input variables as well as the inherent variability associated with the course of infection in each animal and the spread within the group are considered in the model. Parameter distributions for the disease spread model were obtained from previous work by USDA: APHIS: VS: CEAH (2012) and shown below in Table 1.

**Table 1: Input parameters and distributions used in the FMD within-herd model in a beef cattle herd**

| **Variable** | **Input Distribution/Value** |
| --- | --- |
| **Latent Period (1/ λ1)** | Exponential (0.709) |
| **Pre-clinical Period (1/ λ2)** | Log normal (0.862, 0.774) |
| **Clinical Period (1/ ɣ)** | Gamma (4.752, 0.736) |
| **Carrier Period (1/Tau)** | Normal (1095, 180) |
| **q** | 0.5 |
| **Farm Size** | 10,000 (beef) |
| **Adequate Exposures per time step** | Poisson (1.5) |

The assumptions applied to the model included the following:

- Transmission occurs
- Pre-clinically and clinically infectious animals are equally infective with respect to transmitting FMD.
- Cattle in the susceptible state in a given time period all have an identical probability of becoming infected in the next period (i.e. differences in exposure due to grouping of cows in pens is not considered). This may overestimate the number of adequate exposures in large feedlots.
- Variability in adequate contact due to differences in animal density (number of cattle per unit area) is not considered (i.e. transmission is modeled as frequency dependent).
- The probability that a susceptible animal has an adequate exposure with at least one infected cow during a time step is,

$$P_{t}=1-e^{-\frac{k*N_{I,t}}{N-1}}$$

Where *k* is the number of adequate exposures per infected animal, *N_I,t_* is the number of infectious animals at time *t*, and *N* is the total population size. This equation assumes that the number of adequate contacts each animal has in a time period is Poisson distributed with mean *k*. The number of susceptible animals that become infected with FMD during each time step is,

$$I_{t,t+1}^{new}\sim Binomial(S,P_{t})$$

- Inputs for the analysis are based on published literature and the best current knowledge of the disease biology.

**Model outputs by scenario**

***Scenario 1: The disease is allowed to progress through an infected herd and at least 42 days have passed since the day clinical signs were initially detected prior to movement of asymptomatic cattle at or near target market weights to harvest.***

Scenario 1 estimates recovery for an individual animal at 42 days. The model results show that waiting an additional 6 days for a large herd would result in a lower likelihood of disease transmission from infected animals (Table 2). The model predicts that approximately 46% (4,980/10,000) of recovered cattle will become carriers and FMDv may persist in the pharynx at the time of transportation and the remainder of the cattle will fully recover with no virus persistence.

**Table 2:** **Average number of cattle out of a 10,000 head herd at each disease phase with waiting periods of 42 days after premises disease detection (59 days post-disease introduction to premises) and 48 days post-detection (65 days post-disease introduction)**

|  | **Days post-disease detection**  **Mean (95% CI)** | |
| --- | --- | --- |
| **Disease phase** | **42 days** | **48 days** |
| Susceptible | 372(255-489) | 372(255-489) |
| Latent | 0.19(0.12-0.26) | 0 |
| Pre-clinically infectious | 1.17(0.68-1.65) | 0.002(-.0008-0.005) |
| Clinically infectious | 188(177-199) | 0.94(0.86-1.02) |
| Recovered | 4784(4725-4843) | 4,980(4,919-5,040) |
| Carrier | 4654(4598-4711) | 4,648(4,778-4,835) |

***Scenario 2: The feedlot is actively infected (animals with clinical signs are present) and cattle not showing clinical signs of FMD (non-infected, latent, viremic non-clinical, recovered) that are at or near target market weights are moved to harvest without a waiting period.***

Depending on where the transportation date falls in the progression of the disease through the feedlot, there could be a higher or lower likelihood of disease transmission associated with the movement of the animals (see Table 3). In addition, a feedlot that is currently infected is likely to contain more virus in the environment, compared to a recovered feedlot or a feedlot close to recovery, due to the presence of viremic animals that are actively shedding virus. For these reasons, transporting cattle in Scenario 2 will result in a higher likelihood of transmitting FMDv to susceptible animals than transporting cattle in Scenario 1 (unvaccinated cattle - waiting period of 42 days post-detection).

**Table 3:** **Average number of cattle out of a 10,000 head herd at each disease phase at 0, 11, 18, 25, 32, and 39 days after premises disease detection when asymptomatic cattle are moved as soon as harvest eligible (no waiting period such as in Scenario 1)**

|  | **Days post-disease detection**  **Mean (95% CI)** | | | | | |
| --- | --- | --- | --- | --- | --- | --- |
| Disease phase | **0 days** | **11 days** | **18 days** | **25 days** | **32 days** | **39 days** |
| Susceptible | 6,620  (6,547-6,694) | 517  (396-638) | 377  (260-494) | 372  (255-489) | 372  (255-489) | 372  (255-489) |
| Latent | 1,376  (1,319-1,432) | 123  (102-144) | 6.3  (2.4-10.2) | 0.2  (0.1-0.3) | 0.03  (0.02-0.05) | 0.006  (0.0005-0.01) |
| Pre-clinically infectious | 1,095  (1,064-1,125) | 512  (470-554) | 31  (22-39) | 1.2  (0.7-1.6) | 0.1  (0.06-0.2) | 0.02  (0.01-0.03) |
| Clinically-infectious | 692  (682-702) | 2,851  (2,783-2,918) | 861  (823-900) | 188  (177-199) | 38  (36-41) | 7.6  (7.1-8.1) |
| Recovered | 108  (106-111) | 3,010  (2,943-3,077) | 4,397  (4,339-4,456) | 4,784  (4,725-4,843) | 4,891  (4,831-4,950) | 4,937  (4,876-4,997) |
| Carrier | 108  (106-111) | 2,988  (2,922-3,054) | 4,328  (4,271-4,385) | 4,654  (4,598-4,711) | 4,699  (4,642-4,757) | 4,684  (4,627-4,741) |

***Scenario 3: Upon detection, all cattle in the infected feedlot are vaccinated, at least 42 days have passed since the day clinical signs were initially detected in the herd and asymptomatic cattle at or near target market weights are subsequently moved to harvest.***

Relevant values are reported in Table 2, column 2 (42 days post infection)

***Scenario 4: Upon detection, all cattle in the infected feedlot are vaccinated, at least 14 days have passed as the waiting period post-vaccination and cattle not showing clinical signs of FMD (non-infected, latent, viremic non-clinical, recovered) that at or near target market weights are moved to harvest.***

If FMDv was detected on day 17 and cattle were immediately vaccinated, this scenario would mean movement of these animals no sooner than 31 days after initial infection with the 14-day post-vaccination waiting period. Table 4 shows that on day 31, approximately 0.34% (34/10,000) of the herd will be in the latent phase, and approximately 1.59% (159/10,000) of the herd will be in the pre-clinically infectious phase. Should a decision be made to move the eligible cattle as stated in this scenario, a higher likelihood of disease transmission would be present than in Scenarios 1 (unvaccinated cattle – waiting period of 42 days post-detection) and 3 (vaccinated cattle – waiting period of 42 days post-detection) where there would be smaller chance of moving pre-clinical cattle due to the waiting periods in those scenarios (Table 4).

**Table 4: Number of cattle in each disease phase out of a 10,000 head herd at 14 days after premises disease detection (and 14 days post-vaccination)**

| **Disease Phase** | **Days post-disease detection**  **Mean (95% CI)** |
| --- | --- |
| Susceptible | 317 (214-420) |
| Latent | 34 (22-46) |
| Pre-clinically infectious | 159 (138-179) |
| Clinically-infectious | 1862 (1802-1922) |
| Recovered | 3836 (3779-3894) |
| Carrier | 3792 (3735-3849) |

***Scenario 5: The feedlot is not known to be infected (infected but undetected or negative) and is located within a Control Area. All animals have been vaccinated and cattle at or near target market weights are moved to harvest after a 14-day waiting period.***

Assuming the initial infection occurred less than 17 days prior to the date of movement, the disease would therefore go undetected. For example, should a decision be made to move animals 14 days after the herd was infected, there is a higher likelihood of transporting a large number of viremic pre-clinical animals as approximately 3% (343/10,000) of the herd would fall in this category. Table 5 summarizes the number of animals that can be expected to be in each phase during day 14.

If the herd remains uninfected, there would be no risk to nearby susceptible premises during movement of these cattle.

**Table 5: Number of cattle in each disease phase out of a 10,000 head herd at 14 days post-disease introduction to premises**

| **Disease Phase** | **Days post-disease introduction**  **Mean (95% CI)** |
| --- | --- |
| Susceptible | 8,954 (8,886-9,022) |
| Latent | 438 (404-471) |
| Pre-clinically infectious | 343 (318-368) |
| Clinically-infectious | 204 (190-219) |
| Recovered | 31 (29-33) |
| Carrier | 31 (29-33) |
